# Adverse Events During Supervised Exercise Interventions in Pediatric Oncology—A Nationwide Survey

**DOI:** 10.3389/fped.2021.682496

**Published:** 2021-08-19

**Authors:** Gabriele Gauß, Ronja Beller, Joachim Boos, Julia Däggelmann, Hannah Stalf, Joachim Wiskemann, Miriam Götte

**Affiliations:** ^1^Department of Pediatric Hematology and Oncology, Pediatrics III, West German Cancer Center, University Hospital Essen, Essen, Germany; ^2^Department of Pediatric Hematology and Oncology, University Children's Hospital Muenster, Münster, Germany; ^3^Department of Molecular and Cellular Sports Medicine, Institute of Cardiology and Sports Medicine, German Sport University Cologne, Cologne, Germany; ^4^Division of Pediatric Hematology and Oncology, Hospital for Children and Adolescents, University Hospital Frankfurt, Goethe University, Frankfurt am Main, Germany; ^5^Working Group Exercise Oncology Division of Medical Oncology, University Clinic Heidelberg and National Center for Tumor Diseases (NCT), Heidelberg, Germany

**Keywords:** physical activity, childhood cancer, oncological exercise therapy, questionnaire, adverse events

## Abstract

**Objectives:** Exercise interventions during and after treatment for pediatric cancer are associated with beneficial physical, psychological, and social effects. However, valid data about adverse events (AEs) of such interventions have rarely been evaluated. This retrospective study evaluates AEs that occurred during supervised oncological exercise programs for pediatric cancer patients and survivors.

**Methods:** This Germany-wide study used a self-administered online survey focusing on general program characteristics and AEs retrospectively for 2019. The questionnaire included (a) basic data on the offered exercise program, (b) AEs with consequences (Grade 2–5) that occurred in 2019 during an exercise intervention, (c) number of Grade 1 AEs, (d) safety procedures as part of the exercise programs, and (e) possibility to give feedback and describe experience with AEs in free text.

**Results:** Out of 26 eligible exercise programs, response rate of program leaders was 92.3% (*n* = 24). Representatives working for Universities (*n* = 6), rehabilitation clinics (*n* = 3), acute cancer clinics (*n* = 12), and activity camps (*n* = 3) participated. In total, 35,110 exercise interventions with varying duration were recorded for 2019. Six AEs with consequences (Grade 2–3) occurred during exercise interventions after cancer treatment resulting in an incidence of 17 per 100,000 exercise interventions (0.017%). No life-threatening consequences or death were reported and no serious AE occurred during acute cancer treatment. Grade 1 AE occurred with a frequency of 983, corresponding to an incidence of 2,800 per 100,000 interventions (2.8%). Most frequent Grade 1 AE were muscle soreness, circulatory problems, and abdominal pain. The most frequent preventive safety procedures at the institutions were regular breaks, consultations with the medical treatment team, and material selection with low injury potential.

**Conclusions:** Supervised exercise interventions for pediatric cancer patients and survivors seem to be safe and AEs with consequences comparatively rare when compared to general childhood population data. Occurrence of grade 1 AEs was common, however, causality was probably not evident between AEs and the exercise intervention. Future research should standardize assessment of AEs in clinical practice and research, and prospectively register and evaluate AEs that occur in the context of exercise interventions in pediatric cancer patients and survivors.

## Introduction

Survival rates in children and adolescents with cancer have increased over the last decades and researchers and health care providers attempt to improve the quality of survival and reduction of negative side effects during treatment and survivorship (1). In this context, exercise program, are one possible supportive approach to increase health-related quality of life and counteract the disease and treatment related negative effects (2). Although research about the effectiveness of such exercise programs is still limited, first evidence supports positive effects on muscle strength, cardiopulmonary capacity, functional mobility, fatigue, and quality of life (3–6). Furthermore, first evidence supports the hypothesis that a hospital-based exercise program may reduce days of hospitalization and thus costs of the cancer treatment (7). Although programs to promote exercise worldwide are still rare, numbers are increasing. In an international environmental scan, 46 exercise programs have been identified with 46% (*n* = 21) of those located in Germany (8). In Germany, the interdisciplinary Network ActiveOncoKids (NAOK) is working on the expansion and implementation of exercise programs, individualized exercise counseling, qualification of pediatric oncology exercise professionals, and research projects. The evaluation of risks and benefits of exercise programs in children, adolescents, and young adults during and after cancer treatment is one of those research topics identified as relevant in the field of pediatric exercise oncology (9).

In adult cancer patients, the statement “exercise is medicine in oncology (10)” is increasingly being used. However, information about negative events of this medicine “exercise” is deficient. Although feasibility and safety have been described in the current literature (11), a concise evaluation of adverse events (AEs) occurring during exercise programs for children and adolescents with cancer is lacking. Interventional studies evaluating exercise interventions in childhood cancer patients and survivors mostly acknowledge no adverse events (AEs) (12, 13). However, information about the evaluation of such AEs during interventional exercise studies is sparse, not recorded, or not published and no information is available about AEs occurring outside studies during exercise care programs. The purpose of this work is to retrospectively evaluate the frequency and severity of adverse events that occurred during supervised oncological exercise programs for pediatric cancer patients and survivors.

## Methods

This retrospective cross-sectional study was conducted as a national online survey within the NAOK in Germany. Almost all existing pediatric oncology exercise programs in Germany are NAOK members. Ethical approval was granted by the University of Essen and informed consent was obtained from all those who responded to the online surveys.

### Participants

The participants of the survey were identified through NAOK member list screenings, screening of parent's associations with exercise programs (including sailing and climbing groups for example) from homepages and screening of rehabilitation clinics for pediatric oncology. The responsible program coordinators were contacted in June 2020 via email and asked to provide informed consent and to participate in a self-administered online questionnaire. In case of non-response, a first and second reminder was sent out for all participants after 2 weeks each.

### Exercise Programs

Exercise is defined as a “subset of physical activity that is planned, structured, and repetitive and has as a final or an intermediate objective the improvement or maintenance of physical fitness” (14). An exercise program in our study was defined as a voluntary offer for childhood cancer patients or survivors outside of prescribed physiotherapy. An exercise intervention was defined as one unit of exercise completed by one childhood cancer patient or survivor (e.g., a 60 min exercise group with 20 childhood cancer survivors counted as 20 exercise interventions, 6 days of skiing with 5 h of active skiing daily with 10 childhood cancer survivors counted as 300 exercise interventions). Although friends and family members sometimes participated in those exercise programs, they were not included in the analysis. Since duration of an exercise intervention may vary between patients during acute cancer treatment and after cancer treatment, exercise interventions in this study had varying durations.

### Questionnaire and Data Collection

Since no validated questionnaire was available to assess AEs in pediatric exercise oncology, the questionnaire has been developed by (i) information gathering at the NAOK workshop in January 2020, (ii) an interdisciplinary core team critically reviewing the questions and categories of AEs (definition see Table 1) and tested the preliminary questionnaires for feasibility and clearance, and (iii) published data on AEs in adult oncology (15). The questionnaire was administered via an online platform and included (a) basic data on the offered exercise program including number and modalities of exercise interventions in 2019, (b) number of AEs with consequences (e.g., medication, hospitalization) that occurred in 2019 during an exercise intervention with detailed description of what happened, (c) number of other AEs without consequences, (d) safety procedures as part of the exercise program, and (e) possibility to give feedback and describe experience with AEs in free text. To assess grade 1 AEs (AEs without consequences) and preventive safety measures, the author team predefined AEs (e.g., nausea, pain), and safety measures (e.g., medical clearance, warm-up) that were familiar to them from exercise therapy practice. The respondents were asked to indicate the frequency of these items. It was also possible to add events or measures via a free text field. The respondents were encouraged to answer the questions according to their individual records of exercise interventions and AEs that occurred in 2019. The questionnaire is available via https://www.activeoncokids.de/questionnaire/.

**Table 1:** Categorization of adverse events during exercise interventions.

**Term**	**Description**	**Grades**	**Assessment in the questionnaire**
Grade 2–5 AE (with consequences)	Any unfavorable and unintended sign, symptom, or disease temporally associated with the use of a medical treatment or procedure that may or may not be considered related to the physical activity intervention^a^	Grade 2—Minimal, local or non-invasive intervention indicated; limiting age-appropriate instrumental ADL^b^ Grade 3—Medically significant but not immediately life-threatening; hospitalization or prolongation of hospitalization indicated; disabling; limiting self-care ADL^c^ Grade 4—Life-threatening consequences; urgent intervention indicated Grade 5—Death related to adverse event	In 2019, did you ever experience any of the following as a result of an adverse event while playing sports (e.g., shortness of breath, dizziness, fall)? • Death • Vital Intervention • Limited self-care ADL hospitalization or prolongation of hospitalization • Significant delay in adherence to the treatment protocol • Taking medication • Limited age-appropriate instrumental ADL^b^ 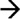 If, yes, detailed description for every event followed
Grade 1 adverse event (without consequences)	Any unfavorable and unintended sign or symptom that is not followed by medical diagnostic or procedure and may or may not be considered related to the physical activity intervention	Grade 1—Clinical or diagnostic observations only; intervention not indicated Shortness of breath, abdominal pain, disorientation, vomiting, hematomas, headache, seizures, circulatory problems, muscle soreness, psychosomatic reaction (e.g., panic attack, outbursts of anger), back pain, abrasions, severe itching, nausea	• Please consider whether those adverse events have occurred and, if so, estimate from your recollection the number that occurred in 2019 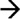 predefined grade 1 AE and free text box for other grade 1 AE

*ADL, Activities of Daily Living; AE, Adverse Event*.

a *Based on CTCAE Common Terminology Criteria for Adverse Events v5.0*.

b *Instrumental ADL refer to preparing meals, shopping for groceries or clothes, using the telephone, managing money, etc*.

c *Self-care ADL refer to bathing, dressing and undressing, feeding self, using the toilet, taking medications, etc*.

To reduce reporting bias, the questionnaire was answered anonymously. However, the participants could provide their contact data voluntarily to answer any queries that would arise.

## Results

**Figure 1:**
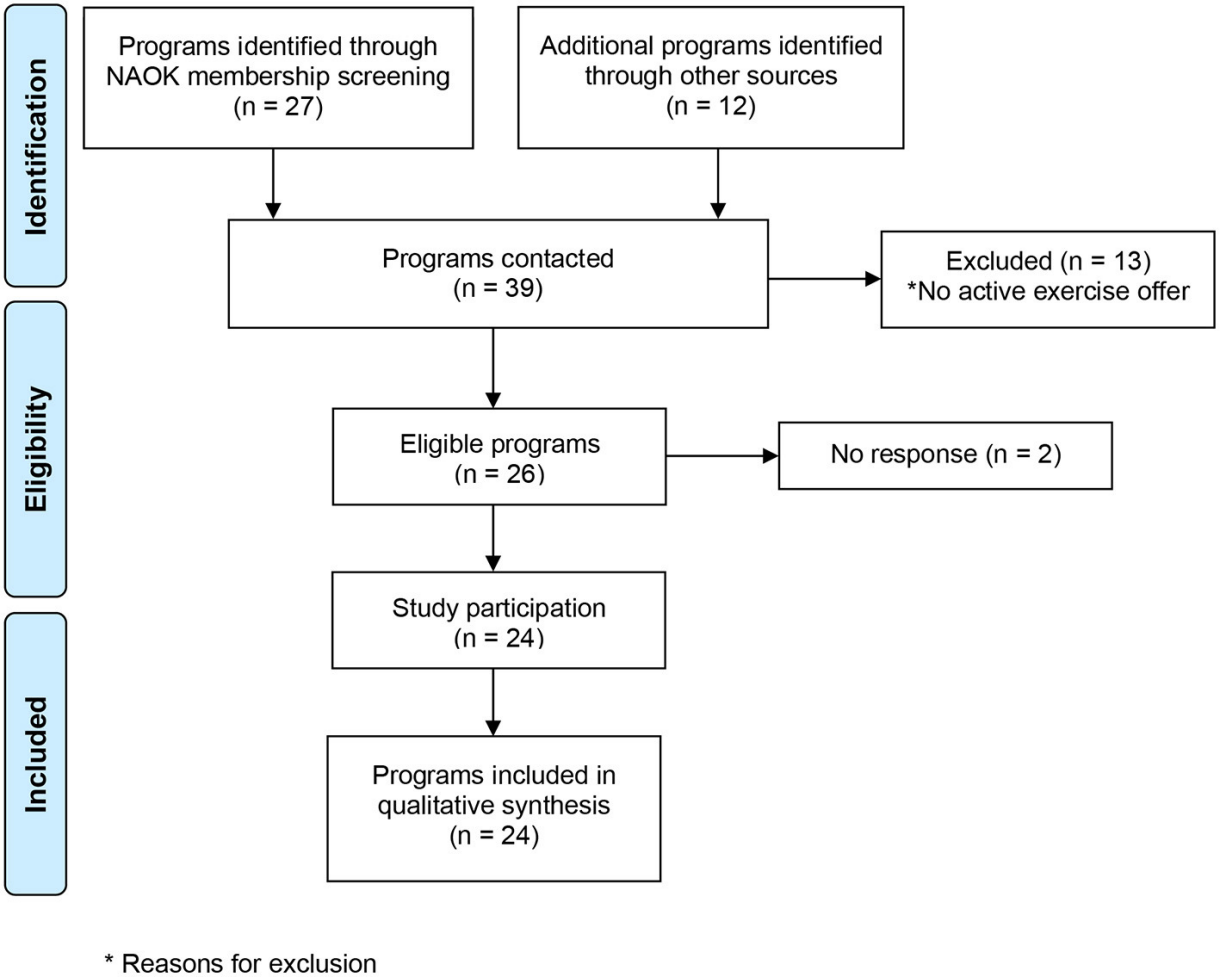
Study recruitment process.

Figure 1 presents a flow diagram of screened and included exercise programs. Out of *n* = 39 identified programs, *n* = 13 organizations were excluded because they had no active exercise offer in 2019. Out of the remaining *n* = 26 eligible programs, *n* = 24 program leaders participated in the study, and *n* = 2 did not participate (response rate 92.3%). Table 2 describes the characteristics of included programs. The included institutions were categorized into rehabilitation clinics (in-patient 4 week rehabilitation based on the legal right to rehabilitation in Germany), activity camps (exercise program offered by non-university institutions, foundations, or commercial organizations), acute cancer clinic (exercise program is implemented into usual care during and/or after cancer treatment and supervised by an exercise professional employed at the pediatric oncology clinic), and university (a university that cooperates with a pediatric oncology clinic to offer an exercise program during and/or after cancer treatment). Based on the information provided by the respondents, the total number of supervised exercise interventions in 2019 was *n* = 35,110 consisting of different modalities of exercise interventions during and after treatment like individual and group sessions in the hospital or rehabilitation camps in the aftercare.

**Table 2:** Characteristics of included facilities (rehabilitation clinics, acute cancer clinics, universities, and activity camps) and their offered exercise programs.

**Possible exercise programs** **in the questionnaire** **(multiple answers possible)**	**Rehabilitation clinics** **(** ***N*** **= 3)**		**Acute cancer clinics** **(** ***N*** **= 12)**		**Universities** **(** ***N*** **= 6)**		**Activity camps** **(** ***N*** **= 3)**		**Total facilities** **(** ***N*** **= 24)**	
	**Number (** ***n*** **) and percentage (%) of facilities offering the listed EX program in 2019**									
	***n***	**%**	***n***	**%**	***n***	**%**	***n***	**%**	***n***	**%**
EX program *during treatment*	0	–	12	100	4	67	1	33	17	71
Inpatient (single/group)	0/0	–	11/3	92/25	4/1	67/17	0/0	–	15/4	63/17
Inpatient (single/group) online	0/0	–	1/0	8/-	0/0	–	0/0	–	1/0	4/–
Outpatient (single/group/home)	0/0/0	–	10/4/0	83/33/-	2/1/0	33/17/–	1/0/0	33/–/–	13/6/0	54/25/–
Outpatient (single/group) online	0/0	–	1/0	8/–	0/0	–	0/0	–	1/0	4/–
EX program *after treatment*	3	100	8	67	6	100	3	100	20	83
Single interventions	2	67	7	58	4	67	1	33	14	54
Group interventions regular^a^	2	67	7	58	2	33	2	67	13	50
Group interventions irregular^b^	0	–	5	42	3	50	3	100	11	42
Group multi-day camps	0	–	3	25	2	33	2	67	7	27
Rehabilitation several weeks	3	100	1	8	2	33	0	–	6	25
Home visits	0	–	0	–	0	–	0	–	0	–
Online (single/group)	0/0	–	0/0	–	0/0	–	0/0	–	0	–
EX interventions in 2019	19,993	57	7,539	22	3,922	11	3,657	10	35,110	100

*EX, exercise*.

a *Exercise program takes place in a defined rhythm (e.g., weekly exercise group)*.

b *Exercise program does not follow a rhythm and take place either once (e.g., ski trip) or selectively throughout the year (e.g., try-out-day)*.

### Adverse Events With Consequences (Grades 2–5)

In total, six AEs occurred that were associated with consequences for the patient in 35,110 exercise interventions (0.02%, incidence 17 per 100,000) with four events being classified as grade 2 and two events as grade 3 [see Table 3, grades 2–5, classification according to CTCAE (16)]. No life-threatening consequences or death (classified as grade 4 or 5) occurred in those 35,110 interventions. All six events occurred during exercise interventions (sport camps, sports group, and rehabilitation) after cessation of acute cancer treatment. One of the six events (line 2, Table 3) did not occur directly during exercising but in the immediate surroundings of the sporting activity during physical activity (walking) and the corresponding environment (ski area). Also, all events involved children and adolescents who were reported to be in average or below average training condition, and all interventions were supervised in a 1:4–1:10 ratio. Table 3 lists all six events with detailed information. Two events (grade 2) resulted in clinical diagnostic and short limitations in some instrumental activities of daily living (ADLs) (e.g., preparing meals, shopping for groceries). Two events resulted in limitations in instrumental ADLs with one being also associated with pain medication. The two highest rated events (grade 3) were both bone fractures and resulted in limitations in instrumental and self-care ADLs. Neither of the two fractures had an indication for surgery. In both grade 3 events, the children were in teenage age and both returned to sports after recovery. Three of the six AEs occurred in the context of skiing. Although the questionnaire does not allow an evaluation of the hours or days per sport, based on the knowledge in the network, it can be assumed that there will be approximately 396 skiing days in 2019, which corresponds to an incidence of 7.6 per 1,000 skiing days.

**Table 3:** Description of occurred Adverse Events with consequences during supervised exercise interventions in pediatric oncology.

	**Patient characteristics**				**Cause of the event**			**Consequences und procedures**				**Grading**
**No**.	**Age,** **years**	**Medical** **background**	**Training** **status**	**Sport** **background**	**What** **happened**	**External** **influence**	**Ratio** **(trainer : child)**	**Type of** **injury**	**Consequences**	**Procedures**	**Other remarks**	**CTCAE** **Grading**
1	15–18	Endoprosthetic device knee (MUTARS), Off-treatment	Below average	Recreational athlete, experienced skier before cancer treatment	Fall while skiing during supervised cancer skiing course on the slope	No visible cause or external influence	1:4–1:6	Contusion	Clinical diagnostic and limitations in some instrumental ADLs	2 days rest	On-site treating surgeon only suggests cycling/swimming as appropriate sports with prosthesis, which confused the teenager	2
2	>18	Endoprosthetic device knee/femoral (MUTARS), Off-treatment	Average	Recreational athlete	Walking accident during skiing camp (tumble on a frozen trail), not during skiing lesson, but in the surrounding area of the sports activity	Snowy and sloping path, limited visibility	1:4–1:6	Heavy pain and ROM limitations	Limitations in instrumental ADLs	14 days of work exemption	Spikes would have been available and recommended by skiing team leader	2
3	10–14	Off-treatment	Average	Recreational athlete	Fall while skiing during supervised skiing course on the slope (last descent)	Pre-riding assistant ski instructor falls; teenager falls down herself while swerving	1:4–1:6	Clavicular fracture and pain	Limitations in instrumental and self-care ADLs (e.g., eating, dressing), prescription of pain medication	Conservative treatment (immobilization arm), no surgery	Incident did not disrupt interest in skiing and sports; re-participation in skiing in the following year	3
4	10–14	Off-treatment	Below average	Without intrinsic motivation for sports, but extrinsic motivation possible	Leg twist on a trampoline during supervised outpatient rehabilitation sports group	No visible cause or external influence	1:4–1:6	Fracture of the lower leg and pain	Limitations in instrumental and self-care ADLs	Plaster cast lower leg, some outpatient clinic visits	Teenager has returned to sports after plaster cast; no longer likes to go on the trampoline (is afraid of twisting again)	3
5	15–18	Off-treatment	Below average	Recreational athlete	Foot twist playing soccer during an inpatient rehabilitation course	No visible cause or external influence	1:7–1:10	Ankle sprain	Limitations in instrumental ADLs, prescription of pain medication	1 week limited participation in activities	Teenager participated in sport activities with modifications the day after the incidence	2
6	6–9	Off-treatment	Average	Recreational athlete	Jumping over a box as part of a sports game during an inpatient rehabilitation course	Time pressure caused by rules of the game, child showed signs of exhaustion previously	1:4–1:6	Straining of ischiocrural muscles	Clinical diagnostic and limitations in some instrumental ADLs	No continuation of the current sport lesson	–	2

*CTCAE, Common Terminology Criteria for Adverse Events; ADL, activities of daily living*.

### Adverse Events Without Consequences (Grade 1)

Grade 1 AEs occurred with a frequency of 983 including muscle soreness (558 without muscle soreness) in the overall 35,110 exercise interventions (2,800 per 100,000 exercise interventions). Frequency and probability of occurrence of the different grade 1 AEs is shown in Table 4. The incidence (cases per 100,000 interventions) varied from 1,208/100,000 (muscle soreness) to 3/100,000 (disorientation). Most events have been classified as uncommon (affects between 1 in 1,000 and 1 in 100 interventions) or rare/very rare (between <1 in 10,000 and 1 in 1,000). Overall probability of any grade 1AEs was rated as common. The most common event was muscle soreness (43.1% of all grade 1 adverse events). Because muscle soreness is not a part of conventional AEs, but can usually occur during exercise, the calculations of frequencies are given inclusive of muscle soreness, and exclusive of muscle soreness.

**Table 4:** Frequency of occurred Grade 1 adverse events during supervised exercise interventions in pediatric oncology.

**Grade 1 adverse event**	***N*** ** (abs.)**	***N*** **(%) of all Grade 1 AEs**		**Incidence (cases Grade** ** 1AE/100,000 interventions)**	**Probability of** ** occurrence^a^**	**Ratio (Grade 1 AE :** ** number of interventions)**
		**Incl. muscle** ** soreness (%/983)**	**Excl. muscle** ** soreness (%/559)**			
Muscle soreness	424	43.1	–	1,208	Common	1:83
Circulatory problems	100	10.2	17.9	285	Uncommon	1:351
Abdominal pain	74	7.5	13.2	211	Uncommon	1:474
Head ache	70	7.1	12.5	199	Uncommon	1:502
Back pain	59	6.0	10.6	168	Uncommon	1:595
Nausea	55	5.6	9.8	157	Uncommon	1:638
Abrasion	49	5.0	8.8	140	Uncommon	1:717
Hematoma	39	4.0	7.0	111	Uncommon	1:900
Psychosomatic reactions	39	4.0	7.0	111	Uncommon	1:900
Itching	33	3.4	5.9	94	Rare	1:1,064
Vomiting	31	3.2	5.5	88	Rare	1:1,133
Dyspnoea	5	0.5	0.9	14	Rare	1:7,022
Seizures	4	0.4	0.7	11	Rare	1:8,776
Disorientation	1	0.1	0.2	3	Very rare	1:35,110
Total (any Grade 1 AEs)	983	100	100	2,800	Common	1:36 incl. and 1:63 excl. muscle soreness

a *According to the side effects classification of medications*.

*N, number; AE, Adverse Event*.

**Figure 2:**
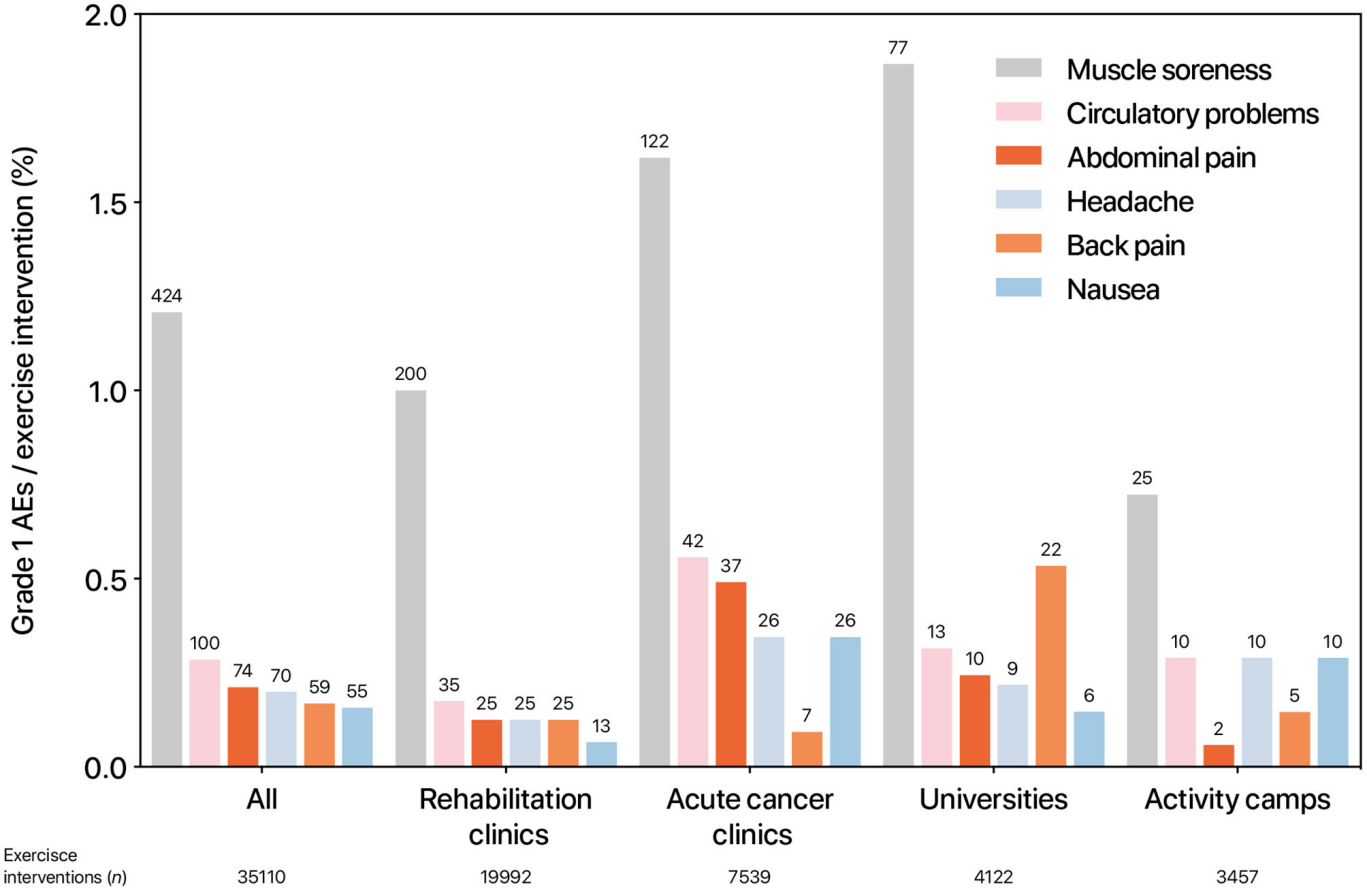
Occurrence of grade 1 adverse events per institution. The bar indicates the relation (%) of performed exercise interventions and adverse events. The numbers above the bars represent the absolute number of events that occurred. AE, adverse events.

In Figure 2, the occurrence (% of offered exercise interventions) of the six most frequent grade 1 AEs (muscle soreness, circulatory problems, abdominal pain, headache, back pain, nausea) are separately presented for rehabilitation clinics, acute cancer clinics, Universities, and activity camps.

### Preventive Safety Procedures

The three most frequent preventive safety procedures at the institutions were regular breaks, consultations with the medical treatment team and appropriate material selection with low injury potential (*n* = 25 answered “always” or “frequent” each). Other frequent procedures (*n* = 23 answered “always” or “frequent” each) were consideration of the subjective state of health of the participants, medical permission, establishing rules of behavior for the child, and a warm-up period. Monitoring of heart rate and or oxygen saturation were only used rarely (*n* = 25 and *n* = 23 answered “rare” or “never”).

## Discussion

This study revealed six AEs grade 2–3 that were associated with the use of a medical treatment or procedure in 35,110 supervised oncological exercise interventions in 2019. All six AEs occurred in exercise programs in the off-treatment phase and no AE was reported for exercise interventions during acute cancer treatment. None of those AEs was fatal, life-threatening, or associated with long-term consequences or lasting impairments. Based on these results it may be stated that exercise interventions for pediatric cancer patients and survivors are safe. However, these six grade 2–3 AEs occurred, which were associated with more or less severe for the patients and although not life-threatening or long-lasting, they compromised the physical and possibly also the psychological condition for a period of time. All children were in average or below-average training condition which allows the conclusion that especially for children who are still physically untrained after the therapy, the re-entry should be well-accompanied and should take place gradually. The two grade 3 events were both bone fractures and resulted in limitations in instrumental and self-care ADLs which is especially unfortunate for young people who have been dependent on help from their parents for so long. At this point, however, it is important to point out that accidents occur during sports. Whether in physical education at school, at club sports, during recreational sports, or even rehabilitation sports, exercise, and playing sports are always associated with the risk of injury (17–19). In comparison to injuries in children in general, the incidence of serious AEs in our cohort is comparably low. The systematic review of Nauta et al. (19) reports that the medically treated injury incidence rate in the included studies with children aged 6–12 years was between 0.15 and 0.56 injuries per 1,000 h of physical activity (vs. 0.17 in our cohort). Nevertheless, it is noteworthy that not one AE graded >1 occurred during the acute treatment phase. Possible reasons could be the lower supervision ratio in aftercare programs, the practice of sports involving risk, or an increase in intensity that is unaccustomed high in comparison with the programs during therapy. Structured rehabilitation training prior to corresponding activities with high intensity after treatment should therefore be considered.

In comparison to the very low number of grade >2 AEs, the overall occurrence of grade 1 AEs was common. However, it is important to consider that in many cases there was probably no causality between the grade 1 AEs that occurred and the exercise intervention, as they were predominantly events that could also be attributed to the medical therapy (i.e., nausea, pain). Other grade 1 AEs like muscle soreness, are a typical sign of intense exercise training, and may occur in healthy as well as diseased children (20). Signs of physical strain (i.e., increased heart rate, physical warming) should be discussed with children and adolescents exercising during cancer treatment as part of normal athletic education. Some children and adolescents even might accept muscle soreness as part of a normality they had before cancer treatment. But if muscle soreness increases individual burden during treatment, the intensity should be reduced. Itching as an AE in the cohort included here, seems to be traced back to vibration exercises (21) that are conducted regularly at one hospital included in this study.

The safety procedures applied within these sites underline the need to communicate with the medical treatment team and the patients and parents (structure and communication), to use low-risk exercise equipment (instruments and material), and to adapt the exercise intervention to the condition of the child (content and individualization).

In comparison to reported AEs in adult oncology (15), the number of overall AEs in our study is slightly higher. Wiskemann et al. reported 672 AEs in 91,252 exercise interventions. Thereof, 10 events were rated as serious AEs (11 out of 100,000 exercise interventions) including bone fractures, scar fractures, torn ligaments, and head injuries, and two cardiac arrest requiring reanimation. Comparing the two studies, we seem to have recorded a higher number of AEs in pediatric oncology, while the severity of AEs was reported to be lower. However, the reasons for these differences may also lie in a differing classification and recording of the events and should be interpreted with caution. Other international research leads into the same direction, that exercise is safe for patients during acute cancer treatment (22), during survivorship (23), and during palliative care (24).

The strength of this study lies in its nationwide multicentric design and the inclusion of the acute as well as the off-treatment phase. Furthermore, different institutions (i.e., rehabilitation clinics and acute cancer clinics) were questioned. A limitation of this study is the retrospective assessment of AEs, which might have led to over- or underreporting of events, especially for grade 1 AEs. However, more serious AEs (grade 2–5) in 1 year are unlikely to be forgotten because they are associated with medical procedures, and a report of the accident. Furthermore, frequencies and types of AEs should be interpreted carefully because the underlying number of interventions varies. The design of the questionnaire does not allow to put number of AEs in relation to conducted number of interventions in a specific sport. As an example, three AEs occurred in the context of skiing. However, we know from network communications and experience that skiing is one of the most offered exercise programs after pediatric cancer treatment and numbers of AEs are probably higher because the total amount of skiing interventions was very high. The estimated incidence of 7.6 AEs per 1,000 skiing days corresponds to the incidence of skiing accidents of 3.9–9.1/1,000 skiing days reported in the literature for children and adolescents in general (25). This is also a common problem in the assessment of AEs during physical education lessons, as written in national reports (26).

Future research should prospectively assess AEs that occur in the context of exercise interventions in pediatric cancer patients and survivors in a nationwide or even international registry. This might not only improve data quality, but also allows for constructive safety warnings to reduce the risk of the same AEs at different institutions. To realize this registry, a standardized definition and classification of AEs are important. From a translational point of view, guidelines that specify existing guidelines (27) by the aspects planning, conducting, monitoring, and evaluation of exercise interventions in pediatric oncology, might help to ensure quality, safety, and efficacy of exercise programs for children and adolescents with cancer.

## Data Availability Statement

The raw data supporting the conclusions of this article will be made available by the authors, without undue reservation.

## Ethics Statement

The studies involving human participants were reviewed and approved by University Duisburg-Essen. Written informed consent from the participants' legal guardian/next of kin was not required to participate in this study in accordance with the national legislation and the institutional requirements.

## Author Contributions

MG, GG, and RB conceptualized and designed the study, collected data, carried out the analyses, drafted the initial manuscript, and revised the manuscript. JB, JD, JW, and HS participated in the conceptualization of the study, provided critical feedback throughout data collection and extraction, and critically reviewed the manuscript for important intellectual content. All authors approved the final manuscript as submitted and agree to be accountable for all aspects of the work.

## Conflict of Interest

The authors declare that the research was conducted in the absence of any commercial or financial relationships that could be construed as a potential conflict of interest.

## Publisher's Note

All claims expressed in this article are solely those of the authors and do not necessarily represent those of their affiliated organizations, or those of the publisher, the editors and the reviewers. Any product that may be evaluated in this article, or claim that may be made by its manufacturer, is not guaranteed or endorsed by the publisher.

## Abbreviations

ADL: Activity of Daily Living
AE: Adverse Event
NAOK: Network ActiveOncoKids.

## References

[1] Langer T Grabow D Steinmann D Wörmann B Calaminus G. Late effects and long-term follow-up after cancer in childhood. Oncol Res Treat. (2017) 40:746–50. 10.1159/000484936 29183026

[2] Kesting S Weeber P Schönfelder M Renz BW Wackerhage H Luettichau IV. Exercise as a potential intervention to modulate cancer outcomes in children and adults? Front Oncol. (2020) 10:196. 10.3389/fonc.2020.00196 32154183 PMC7047207

[3] Senn-Malashonak A Wallek S Schmidt K Rosenhagen A Vogt L Bader P. Psychophysical effects of an exercise therapy during pediatric stem cell transplantation: a randomized controlled trial. Bone Marrow Transplant. (2019) 54:1827–35. 10.1038/s41409-019-0535-z 31089282

[4] Nielsen MKF Christensen JF Frandsen TL Thorsteinsson T Andersen LB Christensen KB. Effects of a physical activity program from diagnosis on cardiorespiratory fitness in children with cancer: a national non-randomized controlled trial. BMC Med. (2020) 18:175. 10.1186/s12916-020-01634-6 32624004 PMC7336676

[5] Morales JS Valenzuela PL Rincón-Castanedo C Takken T Fiuza-Luces C Santos-Lozano A. Exercise training in childhood cancer: a systematic review and meta-analysis of randomized controlled trials. Cancer Treat Rev. (2018) 70:154–67. 10.1016/j.ctrv.2018.08.012 30218787

[6] Stössel S Neu MA Wingerter A Bloch W Zimmer P Paret C. Benefits of exercise training for children and adolescents undergoing cancer treatment: results from the randomized controlled MUCKI trial. Front Pediatr. (2020) 8:243. 10.3389/fped.2020.00243 32582585 PMC7290004

[7] Morales JS Santana-Sosa E Santos-Lozano A Baño-Rodrigo A Valenzuela PL Rincón-Castanedo C. Inhospital exercise benefits in childhood cancer: a prospective cohort study. Scand J Med Sci Sports. (2020) 30:126–34. 10.1111/sms.13545 31482597

[8] Wurz A Daeggelmann J Albinati N Kronlund L Chamorro-Viña C Culos-Reed SN. Physical activity programs for children diagnosed with cancer: an international environmental scan. Support Care Cancer. (2019) 27:1153–62. 10.1007/s00520-019-04669-5 30726517

[9] Wurz A McLaughlin E Chamorro Viña C Grimshaw SL Hamari L Götte M. Advancing the field of pediatric exercise oncology: research and innovation needs. Curr Oncol. (2021) 28:619–29. 10.3390/curroncol28010061 33498499 PMC7924382

[10] Schmitz KH Campbell AM Stuiver MM Pinto BM Schwartz AL Morris GS. Exercise is medicine in oncology: engaging clinicians to help patients move through cancer. CA Cancer J Clin. (2019) 69:468–84. 10.3322/caac.21579 31617590 PMC7896280

[11] Rustler V Hagerty M Daeggelmann J Marjerrison S Bloch W Baumann FT. Exercise interventions for patients with pediatric cancer during inpatient acute care: a systematic review of literature. Pediatr Blood Cancer. (2017) 64:e26567. 10.1002/pbc.26567 28423225

[12] Fiuza-Luces C Padilla JR Soares-Miranda L Santana-Sosa E Quiroga JV Santos-Lozano A. Exercise intervention in pediatric patients with solid tumors: the physical activity in pediatric cancer trial. Med Sci Sports Exerc. (2017) 49:223–30. 10.1249/MSS.0000000000001094 27631396

[13] Saultier P Vallet C Sotteau F Hamidou Z Gentet J-C Barlogis V. A randomized trial of physical activity in children and adolescents with cancer. Cancers. (2021) 13:121. 10.3390/cancers13010121 33401713 PMC7795208

[14] Caspersen CJ Powell KE Christenson GM. Physical activity, exercise, and physical fitness: definitions and distinctions for health-related research. Public Health Rep. (1985) 100:126–31. 3920711 PMC1424733

[15] Clauss D Quirmbach F Wiskemann J Rosenberger F. Adverse Events beim Training mit onkologischen Patienten: Wie sicher ist das Training außerhalb klinischer Studien? Bewegungstherapie und Gesundheitssport. (2019) 35:194–201. 10.1055/a-9057-1883

[16] U.S. Department of Health and Human Services. Common Terminology Criteria for Adverse Events (CTCAE) v5.0. (2017). Available online at: https://ctep.cancer.gov/protocoldevelopment/electronic_applications/docs/CTCAE_v5_Quick_Reference_8.5x11.pdf (accessed March 03, 2021).

[17] Sollerhed A-C Horn A Culpan I Lynch J. Adolescent physical activity-related injuries in school physical education and leisure-time sports. J Int Med Res. (2020) 48:300060520954716. 10.1177/0300060520954716 32967515 PMC7521056

[18] Räisänen AM Kokko S Pasanen K Leppänen M Rimpelä A Villberg J. Prevalence of adolescent physical activity-related injuries in sports, leisure time, and school: the National Physical Activity Behaviour Study for children and Adolescents. BMC Musculoskelet Disord. (2018) 19:58. 10.1186/s12891-018-1969-y 29448928 PMC5815200

[19] Nauta J Martin-Diener E Martin BW van Mechelen W Verhagen E. Injury risk during different physical activity behaviours in children: a systematic review with bias assessment. Sports Med. (2015) 45:327–36. 10.1007/s40279-014-0289-0 25430601

[20] Eston R Byrne C Twist C. Muscle function after exercise-induced muscle damage: considerations for athletic performance in children and adults. J Exerc Sci Fitness. (2003) 1:85–96. Available online at: http://hdl.handle.net/10034/69489 (accessed August 03, 2021).

[21] Rustler V Prokop A Baumann FT Streckmann F Bloch W Daeggelmann J. Whole-body vibration training designed to improve functional impairments after pediatric inpatient anticancer therapy: a pilot study. Pediatr Phys Ther. (2018) 30:341–9. 10.1097/PEP.0000000000000536 30277971

[22] Courneya KS Segal RJ Mackey JR Gelmon K Reid RD Friedenreich CM. Effects of aerobic and resistance exercise in breast cancer patients receiving adjuvant chemotherapy: a multicenter randomized controlled trial. J Clin Oncol. (2007) 25:4396–404. 10.1200/JCO.2006.08.2024 17785708

[23] Dennett AM Peiris CL Shields N Prendergast LA Taylor NF. Moderate-intensity exercise reduces fatigue and improves mobility in cancer survivors: a systematic review and meta-regression. J Physiother. (2016) 62:68–82. 10.1016/j.jphys.2016.02.012 26996098

[24] Heywood R McCarthy AL Skinner TL. Safety and feasibility of exercise interventions in patients with advanced cancer: a systematic review. Support Care Cancer. (2017) 25:3031–50. 10.1007/s00520-017-3827-0 28741176

[25] Meyers MC Laurent CM Higgins RW Skelly WA. Downhill ski injuries in children and adolescents. Sports Med. (2007) 37:485–99. 10.2165/00007256-200737060-00003 17503875

[26] Deutsche Gesetzliche Unfallversicherung e.V., editor. Statistik Schülerunfallgeschehen. Berlin (2019).

[27] Wurz A McLaughlin E Lategan C Chamorro Vina C Grimshaw SL Hamari L. The international pediatric oncology exercise guidelines (iPOEG). Transl Behav Med. (2021) ibab028. 10.1093/tbm/ibab028. [Epub ahead of print]. 34037786 PMC8604278

